# Ten years of collecting hematological athlete biological passport samples—perspectives from a National Anti-doping Organization

**DOI:** 10.3389/fspor.2022.954479

**Published:** 2022-07-19

**Authors:** Lasse V. Bækken, Geir Holden, Astrid Gjelstad, Fredrik Lauritzen

**Affiliations:** ^1^Nordic Athlete Passport Management Unit, Norwegian Doping Control Laboratory, Department of Pharmacology, Oslo University Hospital, Oslo, Norway; ^2^Department of Testing, Investigations and Legal, Anti-doping Norway, Oslo, Norway; ^3^Science and Medicine, Anti-doping Norway, Oslo, Norway; ^4^Section of Pharmaceutical Chemistry, Department of Pharmacy, University of Oslo, Oslo, Norway

**Keywords:** ABP, blood doping, anti-doping, sport, test statistics, ADO, risk assessment, test distribution plan

## Abstract

The hematological module of the Athlete Biological Passport (ABP) aims to reveal blood doping indirectly by looking at selected biomarkers of doping over time. For Anti-Doping Organizations (ADOs), the ABP is a vital tool in the fight against doping in sports through improved target testing and analysis, investigations, deterrence, and as indirect evidence for use of prohibited methods or substances. The physiological characteristics of sport disciplines is an important risk factor in the overall risk assessment and when implementing the hematological module. Sharing of experiences with implementing the hematological ABP between ADOs is key to further strengthen and extend its use. In this study, we present 10 years of experience with the hematological ABP program from the perspectives of a National ADO with special attention to sport disciplines' physiological characteristics as a potential risk factor for blood doping. Not surprisingly, most samples were collected in sport disciplines where the aerobic capacity is vital for performance. The study highlights strengths in Anti-Doping Norway's testing program but also areas that could be improved. For example, it was shown that samples were collected both in and out of season in a subset of the data material that included three popular sports in Norway (Cross-Country Skiing, Nordic Combined, and Biathlon), however, from the total data material it was clear that athletes were more likely to be tested out of competition and on certain days of the week and times of the day. The use of doping control officers with a flexible time schedule and testing outside an athlete's 60 min time-slot could help with a more even distribution during the week and day, and thus reduce the predictability of testing. In addition to promoting a discussion on testing strategies, the study can be used as a starting point for other ADOs on how to examine their own testing program.

## Introduction

The Athlete Biological Passport (ABP) is a vital tool in today's fight against doping in sports. As opposed to traditional anti-doping testing, which aims to directly identify prohibited substances and methods in an athlete's urine or blood sample, the ABP monitors selected variables (biomarkers of doping) over time to indirectly reveal doping by looking for the effects of prohibited substances and methods (Sottas et al., 2011a). By using a Bayesian statistical approach, the ABP calculates individual reference ranges based on both population data and the athlete's own previous results and identify atypical individual changes in the selected biomarker(s) (Sottas et al., 2010; Robinson et al., 2017).

The ABP currently consists of a steroidal and a hematological module, with the latter aiming, according to the World Anti-Doping Agency (WADA), “to identify enhancement of oxygen transport, including use of erythropoiesis-stimulating agents and any form of blood transfusion or manipulation.” The main biomarkers in the hematological module are currently the hemoglobin concentration, reticulocyte percentage, OFF-score, and the abnormal blood profile score (ABPS) (Robinson et al., 2017). The hematological ABP has a wide range of applications, including for example better target testing and analysis (Zorzoli and Rossi, 2012), investigations (Pound, 2015), deterrence (Zorzoli and Rossi, 2010), and as indirect evidence that a prohibited substance or method has been used in accordance with the World Anti-Doping Code Article 2.2 (WADA, 2014). Moreover, the implementation of the hematological ABP by Anti-Doping Organizations (ADOs), herein National ADOs and International Federations (IFs), require a thorough review of risk factors, such as different sport disciplines' physiological requirements and potential performance benefits of blood doping, popularity, and history of blood doping (WADA, 2021a,b).

Publications describing ADOs' practical experience with the implementation of ABP programs provide valuable information that in turn can be used to further optimize ABP programs, as well as for demonstrating transparency (Zorzoli et al., 2014). For example, the International Cycling Union's experience showed that it is important to collect additional samples (i.e., “A” and “B” ABP blood, blood serum and/or urine) together with hematological ABP samples so that traditional direct anti-doping analyses can be performed in case the biological profile is suspicious (Zorzoli and Rossi, 2010).

Anti-Doping Norway (ADNO) has been collecting blood samples from athletes for individual monitoring of hematological variables since 2004. Data on samples collected since late 2009 has been available in WADA's Anti-Doping Administration and Management System (ADAMS). In this study, we present accumulated testing data from a decade of collecting hematological ABP samples from the perspective of a National ADO with special attention on the sport disciplines' physiological characteristics as a potential risk factor for blood doping.

## Materials and methods

The study comprises data on samples from the beginning of 2010 until the end of 2019. The data was extracted from WADA's ADAMS using the sample collection report and the biological result report. In addition, a minor dataset with samples from 2010, which was not uploaded to ADAMS due to national data restrictions at that time, was added to the material. As the data was used to examine both test statistics and blood biomarker values (the latter in a separate study), it included information on the athlete [e.g., biological passport ID (BPID), gender, date of birth, nationality, sport nationality, athlete level, and sport discipline], the test (e.g., sample code, testing authority, result management authority, date and time of collection, if it was collected in- or out-of-competition, and ABP supplementary form information), and transport and analysis [e.g., laboratory, laboratory comment, type of analyzer, date and time of analysis, blood stability score (BSS) (if available), sample validity, and hematological variables]. The complete data material was checked for duplicates using the sample codes and was handled in Microsoft Excel and IBM SPSS Statistics.

Only samples that had a valid BPID in ADAMS were included. Samples with other testing authority and/or results management authority than ADNO were excluded. Further, samples collected from athletes with both nationality and sport nationality other than Norwegian were excluded, meaning that the study population included Norwegian athletes performing their sport in Norway and abroad (*n* = 5,436 samples from 814 athletes), as well as athletes from other countries participating in sports organized under a Norwegian Sports Federation (*n* = 19 samples from 8 athletes) and Norwegian athletes with dual citizenship (*n* = 61 samples from 8 athletes). After exclusion, the data material contained 5,516 samples from 830 athletes. Of these, ADNO was the sample collection authority for 94% (*n* = 5,198 samples) and all but seven samples were collected with no advance notice.

Anti-Doping Norway has testing authority over approximately two million members of the Norwegian Olympic and Paralympic Committee and Confederation of Sports. The pool of athletes under ADNO's testing authority is defined in three groups according to WADAs International Standard for Testing and Investigation (ISTI) (WADA, 2021a). During the period 2010–2019, 2000–4000 athletes was at a given time defined as *National Level* (NL) athletes. These athletes compete at the highest level nationally. Within the group of NL athletes, between 100 and 150 athletes have each year during the 10-year period been under the highest requirement of providing whereabouts information. This group of athletes is called *Registered Testing Pool* (RTP) athletes and is subjected to the greatest amount of testing. Athletes under ADNO's testing authority who are neither RTP athletes nor NL athletes are defined as *Recreational* athletes.

Sports and sport disciplines, as defined by WADA (WADA, 2020), were categorized into groups based on their physiological characteristics according to ADNO's assessment (Table 1).

**Table 1:** Categorization of sport disciplines included in Anti-Doping Norway's hematological ABP program in the period 2010–2019 based on physiological characteristics.

**Physiological classification group**	**Physiological characteristic(s)**	**Sport disciplines**
VO_2max_ endurance	Performance is defined by the time an athlete uses to cover a relatively long, defined distance. The ability to move at a high pace and maintain a high intensity for the duration of the race is crucial.	*Aquatics*: Swimming Middle Distance 200–400 m, Swimming Long Distance 800 m or greater *Athletics*: Middle Distance 800–1,500 m, Long Distance 3,000 m or greater *Biathlon*: All Disciplines *Canoe/Kayak*: Long Distance 1,000 m *Cycling*: Mountain Bike, Road *Orienteering*: Foot Orienteering, Ski Orienteering *Rowing*: All Disciplines *Skating*: Speed Skating >1,500 m *Skiing*: Cross-Country, Nordic Combined *Triathlon*: All Disciplines
Muscular endurance	Performance is defined as the ability to accelerate the body over a short distance and maintain the maximal power output over a limited time period. Challenge the maximum force-generating capacity, yet the duration is long enough that anaerobic and aerobic capacity becomes an important performance-limiting factor.	*Aquatics*: Swimming Sprint 100 m or less *Athletics*: Combined Events Cycling: BMX *Skating*: Speed skating 1,500 m or less *Skiing*: Alpine
Ball and team	Sports with complex physiological profiles which to a different extent may require endurance, strength, coordination and flexibility. In addition, physiological demands vary within a team depending on each athlete's field position and type of work performed.	*Handball*: All Disciplines *Football*: All Disciplines *Tennis*: All Disciplines
Combat	Sports where two individuals fight each other in time set intervals, where the winner is the athlete who scores the most points or knocks out his or her opponent. Complex physiological profiles where the total duration is similar to some endurance sports, the intensity is similar to the power sports, and the work intervals are similar to many team sports.	*Boxing*: All Disciplines *Judo*: All Disciplines *Kickboxing*: All Disciplines *Taekwondo*: All Disciplines *Wrestling*: All Disciplines
Strength and power	Disciplines with relatively short duration and high intensity. The goal in these sports is to either accelerate the body over a short distance as quickly as possible or accelerate an object as fast and far as possible. Also include powerlifting.	*Athletics*: Jumps, Sprint 400 m or less *Powerlifting*: All Disciplines

Only one athlete level, sport, sport discipline, and physiological category was registered for each sample. However, as the athlete level, sport and/or sport discipline may change during a career, several athletes were registered with more than one athlete level, while some athletes were registered with more than one sport, sport discipline, and/or physiological classification group.

In-competition samples refer to samples collected upon conclusion of the competition (i.e., the athlete was chaperoned after finish of the competition until the sample was collected).

## Results

### Athlete population and test distribution

Of the 5,516 hematological ABP samples collected from 830 athletes in the period 2. January 2010 to 28. December 2019, men constituted 72% of all samples (*n* = 3,972) and all athletes (*n* = 597). A similar gender distribution was seen when separating by athlete level as defined at the time of sample collection; men constituted 71% of all samples and 68% of all athletes at the RTP level, 73% of all samples and athletes at the national level, and 74% of all samples and athletes at the recreational level.

At the time of sample collection, the mean athlete age was 26.6 years (*SD* = 5.2), with a range of 15.6–60.6 years. A two-sided, independent sample *T* test revealed no age difference between males (*M* = 26.7, *SD* = 5.1) and females (*M* = 26.6, *SD* = 5.4) (*p* = 0.87).

Most of the samples were collected from athletes registered as RTP at the time of sample collection (62.6%, *n* = 3,454) followed by NL athletes (32.9%, *n* = 1,815) and Recreational athletes (4.5%, *n* = 247) (Table 2). The VO_2max_ endurance group made up 84.7% of the total samples (*n* = 4,670), followed by Muscular Endurance (8.0%, *n* = 439), Ball and Team (4.9%, *n* = 268), Combat (2.1%, *n* = 117), and Strength and Power (0.4%, *n* = 22) (Table 2). Samples from RTP athletes made up most of the samples within all categories, except in the Ball and Team group. Football and Handball were the only sports with no RTP athletes. Of all samples collected at the recreational level, 93.5% were from the VO_2max_ endurance group.

**Table 2:** Number of hematological ABP samples collected in 2010–2019 by physiological classification and athlete level.

**Physiological classification group**	**Athlete level**	**Samples [*****n*** **(%)]**	
VO_2max_ endurance	*Total*	*4,670 (84.7%)*	
	RTP		3,011 (64.5%)
	NL		1,428 (30.6%)
	Recreational		231 (4.9%)
Muscular endurance	*Total*	*439 (8.0%)*	
	RTP		329 (74.9%)
	NL		99 (22.6%)
	Recreational		11 (2.5%)
Ball and team	*Total*	*268 (4.9%)*	
	RTP		7 (2.6%)
	NL		260 (97.0%)
	Recreational		1 (0.4%)
Combat	*Total*	*117 (2.1%)*	
	RTP		95 (81.2%)
	NL		21 (17.9%)
	Recreational		1 (0.9%)
Strength and power	*Total*	*22 (0.4%)*	
	RTP		12 (54.5%)
	NL		7 (31.8%)
	Recreational		3 (13.6%)
Total	*Total*	*5,516 (100%)*	
	RTP		3,454 (62.6%)
	NL		1,815 (32.9%)
	Recreational		247 (4.5%)

*RTP, Registered testing pool; NL, National level*.

Skiing was the most frequent tested sport (*n* = 1,687), followed by Cycling (*n* = 953), Biathlon (*n* = 558), and Athletics (*n* = 531) (Table 3). These were the only sports with more than 500 samples. Within these sports, Cross-Country Skiing (*n* = 1,326), Road Cycling (*n* = 822), and Athletics Long- and Middle Distance (*n* = 473) were the disciplines with the highest number of samples collected (Table 3). In Biathlon there is only one discipline.

**Table 3:** Number of hematological ABP samples collected in 2010–2019 by sport and discipline.

**Sport**	**Discipline**	**Samples** ***% (n)***
Aquatics	Aquatics total	4.5% (247)
	*Long Distance ≥800 m*	*14.6% (36)*
	*Middle Distance 200–400 m*	*29.6% (73)*
	*Sprint ≤ 100 m*	*55.9% (138)*
Athletics	Athletics total	9.6% (531)
	*Combined Events*	*9% (48)*
	*Jumps*	*0.4% (2)*
	*Long Distance ≥3,000 m*	*53.1% (282)*
	*Middle Distance 800–1,500 m*	*36% (191)*
	*Sprint ≤ 400 m*	*1.5% (8)*
Biathlon	Biathlon total	10.1% (558)
Boxing	Boxing total	0.5% (28)
Canoe/Kayak	Long Distance 1,000 m	3.2% (178)
Cycling	Cycling total	17.3% (953)
	*BMX*	*0.4% (4)*
	*Mountain Bike*	*13.3% (127)*
	*Road*	*86.3% (822)*
Football	Football total	3.2% (175)
Handball	Handball total (Indoor)	1.6% (86)
Orienteering	Orienteering total	4.2% (231)
	*Orienteering*	*94.8% (219)*
	*Ski Orienteering*	*5.2 % (12)*
Powerlifting	Powerlifting total	0.2% (12)
Rowing	Rowing total	3.8% (208)
Skating	Skating total	7.4% (409)
	*Speed Skating ≤ 1,500 m*	*35% (143)*
	*Speed Skating >1,500 m*	*65% (266)*
Skiing	Skiing total	30.6% (1,687)
	*Alpine*	*6.3% (106)*
	*Cross-Country*	*78.6% (1,326)*
	*Nordic Combined*	*15.1% (255)*
Triathlon	Triathlon total	2.1% (117)
Wrestling	Wrestling total	1.1% (59)
Sports with samples from <2 athletes		0.6% (37)

### Number of samples per athlete

The number of samples per athlete differed between physiological classification groups. As a group, Muscular Endurance athletes had most samples per athlete [*median* = 5, *interquartile range (IQR)* = 11], followed by Ball and Team (*median* = 4, *IQR* = 2), VO_2max_ endurance (*median* = 2, *IQR* = 6), Combat (*median* = 2, *IQR* = 7), and Strength and Power (*median* = 1, *IQR* = 1) (Table 4). If only including RTP level samples, the median (IQR) was 10 (9), 9 (18), 6 (13), and 2 (1) for the Muscular Endurance, VO_2max_ endurance, Combat, and Strength and Power groups, respectively. The VO_2max_ endurance group was the group with most athletes with 3 samples or more and the only group with athletes having 40 samples or more (*n* = 21 athletes), as well as the group with the highest number of samples from one athlete (*n* = 58 samples) (Table 4).

**Table 4:** Number of hematological ABP samples per athlete collected in 2010–2019 by physiological classification.

		**Samples**			**Number (proportion) of athletes with** ***n*** **sample(s)**					
**Physiological classification group**	**Athletes (** ***n*** **)**	**Median**	**IQR**	**Max**	**1 sample**	**2**	**3–9**	**10–19**	**20–39**	≥**40**
VO_2max_ endurance	660	2	6	58	292 (44%)	79 (12%)	151 (23%)	58 (9%)	59 (9%)	21 (3%)
Muscular endurance	57	5	11	33	15 (26%)	8 (14%)	15 (26%)	12 (21%)	7 (12%)	
Ball and team	83	4	2	7	14 (17%)	7 (8%)	62 (75%)			
Combat	25	2	7	14	11 (44%)	3 (12%)	6 (24%)	5 (20%)		
Strength and power	14	1	1	3	9 (64%)	2 (14%)	3 (21%)			
Total athletes	839									

### Evolution of the hematological ABP program 2010–2019

The hematological ABP program alone did not result in any anti-doping rule violations in the period examined.

Between 66 and 76% of the annual samples were taken from male athletes, with no clear trend over the years regarding the gender distribution. Throughout the 10-year period more than half of the annual samples were collected from RTP athletes, except in 2013 and 2014, when RTP samples constituted 48 and 47%, respectively.

When examining the number of samples collected per year including all sports, there was an upward trend from 399 samples in 2010 to a peak of 773 samples in 2016 before a gradual downward trend to 538 samples in 2019 (Table 5). Out-of-competition samples constituted 96.2% of the total collected samples, with a yearly range of 82.7–100% during the period. In-competition samples only exceeded 5% of the annually collected samples in 2013 (17.3%) and 2019 (8.2%). In total, 51% of the in-competition samples were collected from athletes at the recreational level.

**Table 5:** Annually collected hematological ABP samples by test type.

**Year**	**Total samples**	**Type of test [% (** ***n*** **)]**	
		**INC**	**OOC**
2010	399	2.8% (11)	97.2% (388)
2011	305	0% (0)	100% (305)
2012	422	0.5% (2)	99.5% (420)
2013	568	17.3% (98)	82.7% (470)
2014	535	2.8% (15)	97.2% (520)
2015	557	4.3% (24)	95.7% (533)
2016	773	1.2% (9)	98.8% (764)
2017	737	0.3% (2)	99.7% (735)
2018	682	0.3% (2)	99.7% (680)
2019	538	8.2% (44)	91.8% (494)
Total	5,516	3.8% (207)	96.2% (5,309)

*INC, In-competition; OOC, Out-of-competition*.

**Figure 1:**
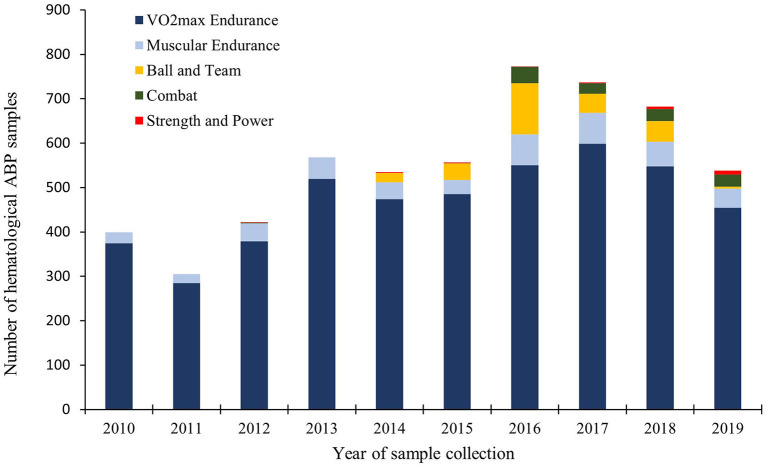
Annually collected hematological ABP samples by physiological classification group.

In the first two years (2010–2011), samples were only collected in VO_2max_ endurance and Muscular Endurance sport disciplines (Figure 1). From 2016 athletes from all five physiological classification groups were tested annually.

Between 61 and 73% of the annual samples in the 10-year period were collected from athletes in Skiing, Cycling, Biathlon and Athletics. Skiing was the most tested sport in all years of the examined period with 105–245 samples collected annually, comprising 26–34% of the annually collected samples.

**Figure 2:**
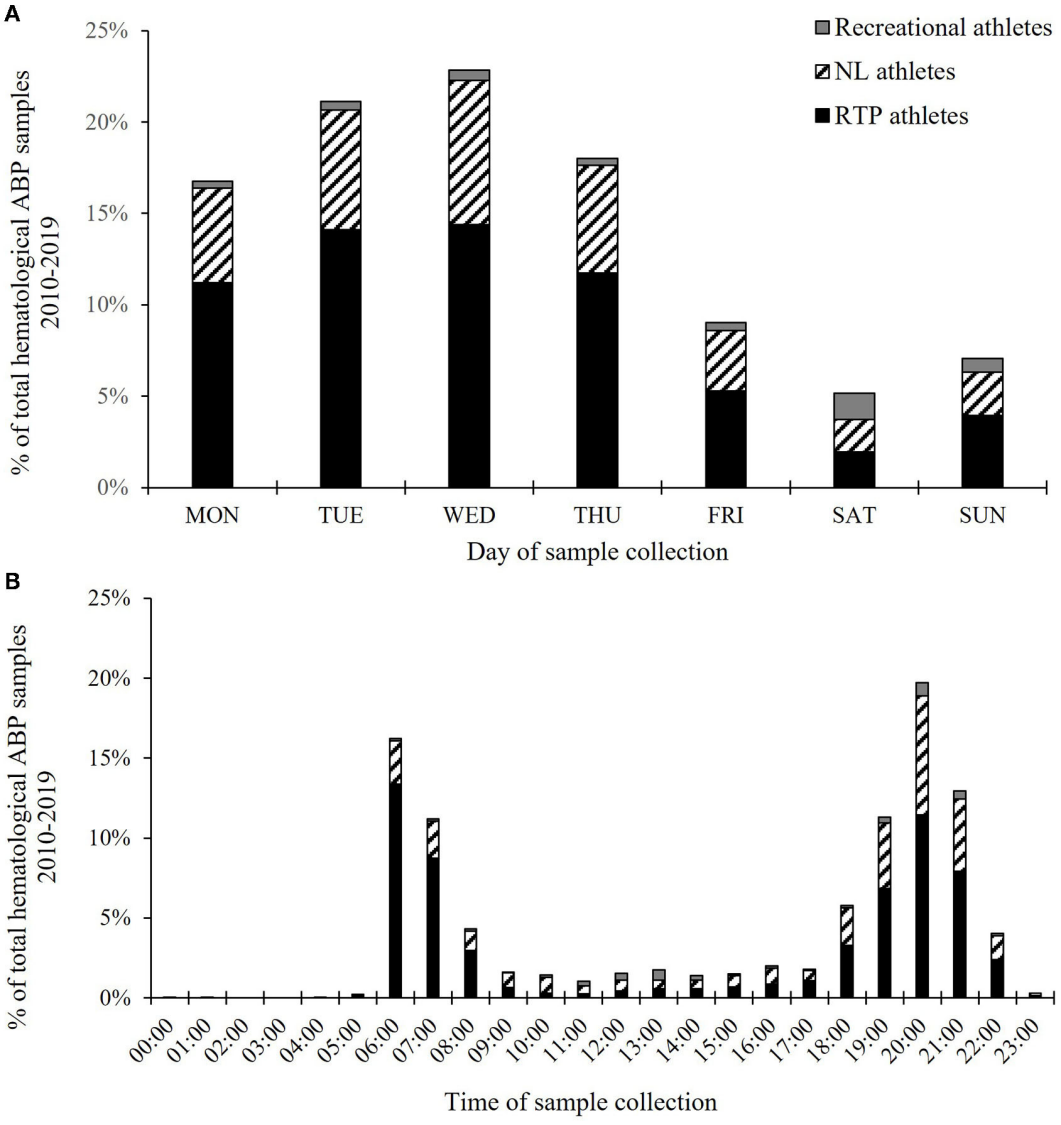
Distribution of hematological ABP samples from Monday to Sunday **(A)** and during the day **(B)** in the period 2010–2019. RTP, Registered testing pool; NL, National level. Time of day data available for 5,491 samples.

When examining the time of testing, most of the samples were collected mid-week (Wednesday: 22.8% and Tuesday: 21.1%), followed by Thursday (18.0%) and Monday (16.8%), while fewest were collected on Friday (9.0%) and during the weekend (Sunday: 7.1% and Saturday: 5.2%) (Figure 2A). There was some variation over the years, however, the pattern was the same every year throughout the 10-year period. Registered Testing Pool and NL athletes had 9.4 and 12.6% of their samples collected during the weekend, respectively. In contrast, 49.4% of the samples collected from Recreational athletes were collected during the weekend.

Samples were collected throughout day and night, with the highest number of samples collected in the time period 20:00–20:59 (*n* = 1,082 samples, 19.7%, Figure 2B). The time of sample collection had a clear bimodal distribution, with peaks in the morning (6:00–8:59, *n* = 1,741 samples, 31.7% of all samples) and evening (18:00–22:59, *n* = 2,952 samples, 53.8% of all samples). Albeit some variation over the years, no year had <78.9% of the samples collected in these two time periods combined. RTP athletes had 90.8% of the group's total samples collected between 6:00–8:59 and 18:00–22:59, while 79.7% and 52.6% of the samples from the NL and Recreational groups were collected in the same time periods, respectively.

**Figure 3:**
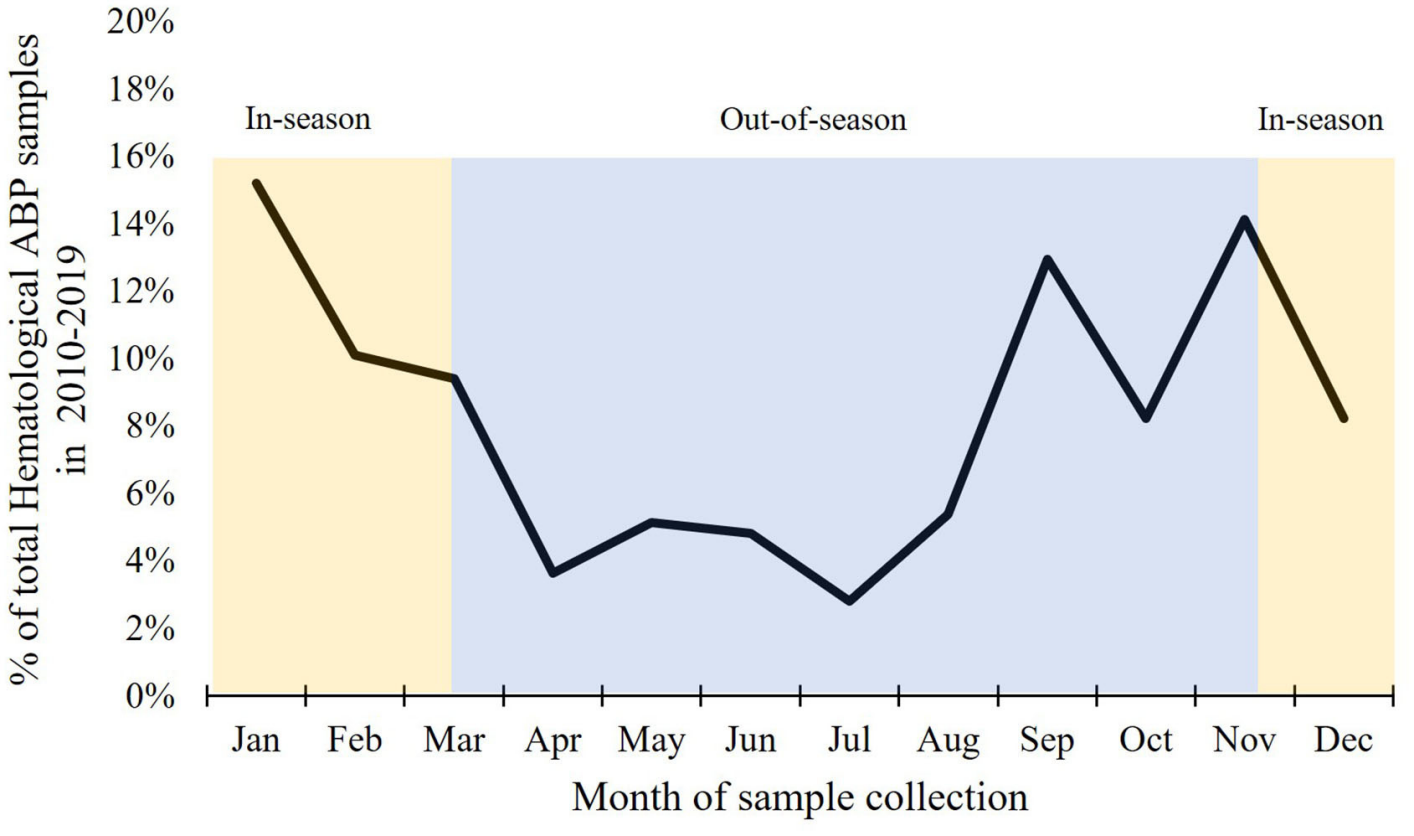
Distribution of samples per month for Biathlon, Cross-Country Skiing and Nordic Combined for the period 2010–2019.

To examine possible patterns in the collection of hematological ABP samples in-season vs. out-of-season, we analyzed Biathlon, Cross-Country Skiing, and Nordic Combined in more detail, as they have similar and clearly defined sporting seasons. Thus, in Figure 3, the relative number of samples per month is shown for a subset of the data material. For the 10-year period in total, January was the month with most samples (325 samples, 15.2%) followed by November (302 samples, 14.1%) and September (277 samples, 12.9%). Fewest samples were collected in April to August. These months collectively had 21.8% of the total number of samples. Over the years, this period's (April-August) relative contribution to the annually collected samples varied between 13.3 to 31.7%.

## Discussion

As expected, the main group for hematological ABP testing was the VO_2max_ endurance group, which is well in line with the sport disciplines' physical requirement of a high aerobic capacity (Joyner and Coyle, 2008; McArdle et al., 2019; van der Zwaard et al., 2021) and potential performance benefits of blood doping (Sgro et al., 2018; Solheim et al., 2019), as well as reported abnormalities in hematological values sensitive to blood doping (Stray-Gundersen et al., 2003; Morkeberg et al., 2009; Zorzoli and Rossi, 2010; Sottas et al., 2011b; Faiss et al., 2020) and blood doping history (Fitch, 2017; Database, 2022) in some of the sport disciplines. A low number of samples per athlete in this group can mainly be explained by testing at the recreational level. This is evident from the number of samples per athlete at the RTP level and by the number of athletes with ten samples or more. Cross-Country Skiing was by far the most tested sport discipline with 24% of all samples collected over the 10-year period. The emphasis on this sport discipline correlates well with its popularity in Norway, as well as the success of Norwegian athletes, illustrated by Norway currently being the all-time most-winning nation at the World Ski Championships and Olympic Winter Games in Cross-Country Skiing (FIS Statistics, 2021). Moreover, using the WADA testing figures since 2012 (necessary details in the testing figures are not available before 2012), ADNO was the National ADO that collected most hematological ABP samples in Skiing from 2014 to 2018 and the National ADO that collected second most in 2012, 2013, and 2019 (WADA, 2022).

Orienteering, another sport in the VO_2max_ endurance group, was the seventh most tested sport with 4.2% of all samples. One could argue, based on the physiological requirement of a high aerobic capacity (Batista et al., 2020), that collection of hematological ABP samples in orienteering is important to detect and deter blood doping. This is however not a requirement by WADA (WADA, 2020). On the other hand, ADNO did not collect hematological ABP samples in some other sport disciplines where aerobic capacity is important, for example cyclo-cross (cycling), track endurance (cycling), and ski mountaineering. One explanation for this is the relative low number of active athletes in Norway.

The second most tested group was the Muscular Endurance group. Like the VO_2max_ endurance group, this group was included in ADNO's hematological ABP program for the whole 10-year period presented in this study. The Muscular Endurance disciplines typically have a shorter duration than those of the VO_2max_ endurance group and are more dependent on muscle strength and anaerobic capacity, however, the duration is still long enough to make the aerobic capacity important for performance (Nemoto et al., 1988; Tesch, 1995; Pyne and Sharp, 2014; McArdle et al., 2019), which in turn could make blood doping attractive. In addition, hemoglobin has a buffering capacity during anaerobic work (Rubana and Aulik, 1989). Swimming sprint 100 m or less was the most tested aquatic sport discipline over the 10-year period in the presented study (138 samples) and constituted 31.4% of all samples in the Muscle Endurance group. For comparison, swimming sprint 100 m or less was the second most tested aquatic sport discipline in the world in 2019 (963 samples), just behind swimming Middle Distance 200–400 m (991 samples) but well ahead of swimming long distance 800 m or greater (384 samples) and open water swimming (292 samples) (WADA, 2022). Although these testing numbers do not take into account the number of participating athletes and other risk factors, it illustrates that blood doping is deemed a risk by ADOs in sprint swimming despite the relative short duration.

The Ball and Team group had the third highest number of samples collected. Performance in these sport disciplines is complex and a certain level of aerobic capacity is important for performance (Manchado et al., 2013; Slimani et al., 2019), thus making blood doping potentially relevant. The introduction of blood sampling for hematological parameters at the 2002 World Cup by the International Federation of Football Associations (FIFA) may illustrate this (Zorzoli et al., 2014). Ball and Team sports were included in ADNO's hematological ABP program in 2014. The highest annual number of ABP blood samples was in 2016 due to ADNO's hematological ABP-project in Handball and Football that year. For Football, the main focus was put on player positions that require a high aerobic capacity (i.e., full-backs and midfielders). To get a good impression of the variation of the hematological markers in each player, most of the athletes were tested at least three times. The hematological results and the reviews of the Athlete Passport Management Unit (APMU) provided ADNO with valuable insight that subsequently led to a reduction in the collection of hematological ABP samples in Handball and Football. This explains why there is a quite high median for the number of samples per athlete in this group, albeit no player had more than five samples. For comparison, it has previously been reported that cyclists had to be tested at least three times over a period of minimum 6 weeks before the rider was allowed to compete at a Union Cycliste Internationale (UCI) World Tour event (Zorzoli et al., 2014). ADOs should consider implementing projects similar to ADNO's Handball- and Football-project to gain insight in sport disciplines where hematological ABP sampling is traditionally not that common for the ADO, which in turn can be used in the overall risk assessment, and thus for developing the overall testing program.

Athletes from the Combat group was first tested in 2012 but has mainly been in the hematological ABP program since 2016. Actually, using WADA's testing figures, ADNO was one of the four most-testing National ADOs in the world in wrestling from 2016 to 2019 (WADA, 2022). Blood doping could be tempting for athletes in such sport disciplines as a high aerobic capacity is important to maintain repetitive high-intensity actions throughout the fight and to accelerate the recovery process between rounds and fights (Franchini et al., 2011; Bridge et al., 2014; Chaabene et al., 2015, 2017). However, for the Combat, as well as the Muscle Endurance and Ball and Team sport disciplines, there is a lack of research investigating potential performance enhancing effects of blood doping. In addition, it has to our knowledge been few (Muscle Endurance and Combat groups) or no (Ball and Team group) athletes sanctioned for blood doping in these sport disciplines (Database, 2022).

Few hematological ABP samples were collected from athletes in Strength and Power sport disciplines. This can reasonably be explained by the fact that performance in these sport disciplines first and foremost is determined by the ability to generate force and power, and therefore blood doping is less attractive. The potential non-erythropoietic ergogenic effects of recombinant EPO, a type of blood doping, should however not be forgotten (Sgro et al., 2018) and a few blood doping cases have been recorded (Database, 2022). Since anabolic androgenic steroids can affect the hematological ABP markers (Mullen et al., 2018; Borjesson et al., 2020; Solheim et al., 2020), it can be interesting to incorporate the hematological ABP in the overall monitoring of some high-risk athletes. It should be noted that about half of the samples collected in athletics sprint 400 m or less were collected from athletes mainly competing in 400 m (with or without hurdles). From a physiological requirement point of view (McArdle et al., 2019), it could be argued that this sport discipline, which is defined by WADA (WADA, 2020), could be divided in “sprint 200 m or less” and “sprint 400 m,” with the latter being classified in the Muscle Endurance group in the present study.

Although the majority of the hematological ABP samples were collected from RTP athletes, the testing of athletes not providing whereabouts is also important. This was recently accentuated when the French Anti-Doping Agency tested at a regional cycling competition in Guadeloupe where 16.6% of the tested athletes were using one or more prohibited substances (Marchand et al., 2017). The testing of NL athletes is important as these athletes are often at a stage in the career where increasing the performance is important to take the step up to the highest level, and therefore may have an increased risk of doping. It should be noted that for Football and Handball, there are no RTP athletes as per ADNO's definition, even if playing at the highest international level. Furthermore, testing at the recreational level may be beneficial for a National ADO in terms of public awareness and deterrence of doping, in addition to detection. In the present study, almost all samples collected at the recreational level were collected in VO_2max_ endurance sports. Furthermore, many of these samples were collected in-competition at big national sporting events during the weekends where many recreational athletes participate. In fact, samples collected at such events made up 72 and 68% of the total number of in-competition samples for all athlete levels in 2013 and 2019, respectively, and largely explains the high number of in-competition tests those 2 years. Mass testing in connection with major events is a strategy that is relevant at all athlete levels, as this makes it possible to efficiently “screen” many athletes and identify certain athletes that needs further attention and target testing, especially those who are not under a regular hematological ABP testing program.

Collecting hematological ABP samples in connection with competitions is crucial as this is when athletes would want to have elevated hemoglobin mass for increased performance. In the 10-year period examined in this study, in-competition samples only constituted 3.8% of all hematological ABP samples collected. One reason for this is that many of the NL- and RTP athletes are under the jurisdiction of the IF when they are competing in international events. In fact, the WADA testing figures show that the relative number of in-competition samples collected by ADNO in 2019 was approximately at the level of the average National ADO (8.3 vs. 7.9%, respectively) (WADA, 2022). In contrast, the relative number of in-competitions samples collected by ADNO was well below the average National ADO in 2018 (0.3 vs. 7.1%, respectively) (WADA, 2022). In comparison, the relative numbers of in-competition samples by IFs (including also other code signatories and sport organizations) were 19.0 and 18.0% for 2018 and 2019, respectively (WADA, 2022). It should be noted that the definition of in-competition samples may differ slightly between ADOs. The collection of in-competition samples by National ADOs as a complement to testing by the IFs is nevertheless important, for example at national championships or important qualifications. It must be emphasized that the definition of an in-competition sample is essential. In the present study, a sample collected the day before or after the competition is defined as an out-of-competition sample, however, it will still likely detect an artificially increased hemoglobin mass (Damsgaard et al., 2006; Haile et al., 2019). On the other hand, an autologous blood transfusion strategy where blood reinfusion is followed by a blood withdrawal on the same day would not be detected with such out-of-competition testing [e.g., as practiced in the doping regime uncovered by the investigation termed “Operation Aderlass” (Hood, 2016)]. In addition to collecting out-of-competition samples within few days before or after major competitions, it is therefore important to collect in-competition samples upon conclusion of the competition (i.e., athlete is chaperoned from finish until the sample is collected), especially if there is suspicion of blood transfusions, such as previous abnormal blood values indicating blood withdrawal, or non-analytical information like tip-offs indicating blood transfusions or sudden extraordinary performance.

To take full advantage of the ABP-tool, samples should be collected throughout the year. For example, due to its negative effects on performance (Malm et al., 2016), blood withdrawal (for later reinfusion) is more likely to be performed out of season far from competitions (red blood cells can be stored up to 30 years if cryopreservation is available) or up to 35–42 days in advance of a competition (if refrigeration is the only option) (EDQM, 2016; Heddle et al., 2016). Further, as the performance enhancing effects of recombinant EPO remains up to several weeks after cessation of use (Durussel et al., 2013), athletes may discontinue the use prior to the competition in order to not test positive (Zorzoli, 2012). Besides, blood doping may be performed to cope with heavy training periods (Faster-Skier, 2019). In addition to testing athletes at the time of competition, it is therefore important to test athletes in the months and weeks leading up to important events during the season, as well as out of season. To study the distribution of hematological ABP samples throughout the year, three sports with similar and clearly defined seasons (Biathlon, Cross-Country Skiing, and Nordic Combined) were selected for further analysis. The results show that samples were in fact collected throughout the year in this group. Although most samples were collected during pre-season and in-season, a fair number of samples were collected during off-season every year.

When studying the timing of sample collection, a clear pattern was identified in that most of the samples were collected during weekdays (Monday-Thursday) and in the morning (6:00–8:59) and evening (18:00–22:59). Although the ABP benefits from the fact that the markers are sensitive also for some time after the excretion of the banned substance or practice of the method (Damsgaard et al., 2006; Haile et al., 2019), it is important that anti-doping testing is unpredictable. This is especially important as ADNO in most cases collect urine and/or blood (“serum”) samples for “direct detection” together with the hematological ABP sample. Hence, even though the effect of a prohibited substance or method may be visible in the ABP markers, the direct detection sensitivity may be lowered for the sophisticated doper. For example, if an athlete over time is only tested during weekdays and in the evening, it may be easier to plan a doping scheme. In this context, it should also be mentioned that testing in the morning may prove beneficial for the detection of low doses of substances with a short half-life used at night (Baumann, 2012). Two possible explanations for the bimodal distribution of sampling throughout the day are the availability of the athletes (e.g., timing of the 60 min time-slots of the RTP athletes and team practice for the Ball and Team sport disciplines) and the availability of the part time doping control officers (tests are often planned and conducted before or after their normal daytime job). More testing of RTP athletes outside the 60-min time slot, although this could result in more missed tests and increased costs, as well as using doping control officers with a flexible time schedule is therefore important to make timing of testing less predictable. Regarding the lower number of samples collected during the weekend, a possible explanation could be that during the competitive season, athletes on the international level are often unavailable for testing by ADNO due to participation in competitions where the IF has the jurisdiction. It is important to emphasize that the observations on timing of testing are on the total data material. For example, after closer investigation we found that in Canoe/Kayak at the national level, it was an even distribution throughout the week with no day with more than 16.9% and no day with less dan 10.2% of the weekly samples. In fact, it is important to examine different testing figures, like timing (year, month, week, day, hour of the day), location, and the use of different doping control officers, in detail when assessing the overall test distribution plan and when planning intelligent testing on certain athlete levels, sport disciplines, teams, and individuals (WADA, 2021a). By doing this, unexpected testing strategies can be implemented.

## Limitations

The main limitation of this study is that the data material only includes samples collected by ADNO. Athletes competing at the international level are also tested by other ADOs with testing jurisdiction, such as IFs and the International Olympic Committee. Further, the data material presented here only includes hematological ABP samples, and it may be that the distribution is different for urine and/or blood (“serum”) samples.

## Conclusion

The present study gives a valuable insight into a National ADO's testing program and how it has developed over a decade.

The hematological ABP testing program of ADNO has evolved from focusing on VO_2max_ endurance and Muscular Endurance sports to also including sports with a more complex physiological profile, such as Ball and Team sports, Combat sports and Strength and Power sports. The inclusion of additional sport disciplines, in particular sport disciplines that require a high aerobic capacity, should however be considered in the future. In addition, it should be considered to collect more hematological ABP samples at the recreational level in different sport disciplines, also outside the VO _2max_ endurance group.

We found that fewer hematological ABP samples were collected during weekends and between 09:00 and 18.00. The use of doping control officers with a flexible time schedule and testing outside an athlete's 60 min time-slot could reduce the predictability of testing. Also, the number of in-competition samples should be increased, as this is important to detect and deter blood doping.

In a subset of the data material, it was shown that hematological ABP samples had been collected throughout the year, also during off season and summer training. This is important as blood withdrawal for later re-infusion might be performed during periods of easy training (i.e., off-season), and because blood doping could be performed to cope with heavy off-season training periods.

The present study may provide inspiration to other ADOs on how to systematically examine and evaluate their own testing program.

## Data availability statement

The data analyzed in this study is subject to the following licenses/restrictions: The dataset consists of anti-doping ABP test statistics that are part of Anti-doping Norway's testing program. Requests to access these datasets should be directed to fredrik.lauritzen@antidoping.no.

## Author contributions

All authors listed have made a substantial, direct, and intellectual contribution to the work and approved it for publication.

## Conflict of interest

The authors declare that the research was conducted in the absence of any commercial or financial relationships that could be construed as a potential conflict of interest.

## Publisher's note

All claims expressed in this article are solely those of the authors and do not necessarily represent those of their affiliated organizations, or those of the publisher, the editors and the reviewers. Any product that may be evaluated in this article, or claim that may be made by its manufacturer, is not guaranteed or endorsed by the publisher.

## Abbreviations

ABP: athlete biological passport
ADAMS: anti-doping administration and management system
ADNO: anti-doping norway
ADO: anti-doping organization
APMU: athlete passport management unit
IF: international federation
NL: national level
RTP: registered testing pool
WADA: world anti-doping agency.

## References

[B1] Batista M. M. Paludo A. C. Gula J. N. Pauli P. H. Tartaruga M. P. (2020). Physiological and cognitive demands of orienteering: a systematic review. Sport Sci. Health 16, 591–600. 10.1007/s11332-020-00650-6

[B2] Baumann G. P.. (2012). Growth hormone doping in sports: a critical review of use and detection strategies. Endocr. Rev. 33, 155–186. 10.1210/er.2011-1035 22368183

[B3] Borjesson A. Lehtihet M. Andersson A. Dahl M. L. Vicente V. Ericsson M.. (2020). Studies of athlete biological passport biomarkers and clinical parameters in male and female users of anabolic androgenic steroids and other doping agents. Drug Test. Anal. 12, 514–523. 10.1002/dta.2763 31925932

[B4] Bridge C. A. Ferreira da Silva Santos J. Chaabene H. Pieter W. Franchini E. (2014). Physical and physiological profiles of taekwondo athletes. Sports Med. 44, 713–733. 10.1007/s40279-014-0159-9 24549477

[B5] Chaabene H. Negra Y. Bouguezzi R. Mkaouer B. Franchini E. Julio U.. (2017). Physical and physiological attributes of wrestlers: an update. J. Strength Cond. Res. 31, 1411–1442. 10.1519/JSC.0000000000001738 28030533

[B6] Chaabene H. Tabben M. Mkaouer B. Franchini E. Negra Y. Hammami M.. (2015). Amateur boxing: physical and physiological attributes. Sports Med. 45, 337–352. 10.1007/s40279-014-0274-7 25358529

[B7] Damsgaard R. Munch T. Morkeberg J. Mortensen S. P. Gonzalez-Alonso J. (2006). Effects of blood withdrawal and reinfusion on biomarkers of erythropoiesis in humans: implications for anti-doping strategies. Haematologica 91, 1006–1008. 16584989

[B8] Database A.-D.. (2022). Anti-Doping Database: Inside Sport AS (2020). Available online at: https://dopinglist.com/ (accessed February 16, 2022).

[B9] Durussel J. Daskalaki E. Anderson M. Chatterji T. Wondimu D. H. Padmanabhan N.. (2013). Haemoglobin mass and running time trial performance after recombinant human erythropoietin administration in trained men. PLoS ONE. 8:e56151. 10.1371/journal.pone.0056151 23418527 PMC3571963

[B10] EDQM (2016). Guide to the Preparation, Use and Quality Assurance of Blood Components. Component monographs Part A and B: European Directorate for the Quality of Medicines and HealthCare of the Council of Europe (2017). *p*. 545.

[B11] Faiss R. Saugy J. Zollinger A. Robinson N. Schuetz F. Saugy M.. (2020). Prevalence estimate of blood doping in elite track and field athletes during two major international events. Front. Physiol. 11:160. 10.3389/fphys.2020.00160 32161553 PMC7052379

[B12] Faster-Skier (2019). Estonia's Karel Tammjärv. Podcast: 1 hour 5 minutes. Available online at: https://fasterskier.com/2019/03/nordic-nation-estonias-karel-tammjarv/ 6 March [accessed cited 7 March 2022].

[B13] FIS Statistics (2021). Available from: https://www.fis-ski.com/DB/general/statistics.html?statistictype=standingsandsectorcode=CC (accessed December 09, 2021).

[B14] Fitch K. D.. (2017). Blood doping at the Olympic Games. J. Sports Med. Phys. Fitness. 57, 1526–1532. 10.23736/S0022-4707.17.06948-1 28094487

[B15] Franchini E. Del Vecchio F. B. Matsushigue K. A. Artioli G. G. (2011). Physiological profiles of elite judo athletes. Sports Med. 41, 147–166. 10.2165/11538580-000000000-00000 21244106

[B16] Haile D. W. Durussel J. Mekonen W. Ongaro N. Anjila E. Mooses M.. (2019). Effects of EPO on blood parameters and running performance in Kenyan athletes. Med. Sci. Sports Exerc. 51, 299–307. 10.1249/MSS.0000000000001777 30188362

[B17] Heddle N. M. Cook R. J. Arnold D. M. Liu Y. Barty R. Crowther M. A.. (2016). Effect of short-term vs. long-term blood storage on mortality after transfusion. N. Engl. J. Med. 375, 1937–1945. 10.1056/NEJMoa1609014 27775503

[B18] Hood A.. (2016). Commentary: What German Anti-Doping Probe May Tell Us About Cycling VeloNews2019. Available online at: https://www.velonews.com/news/commentary-what-german-anti-doping-probe-may-tell-us-about-cycling/ (accessed February 22, 2022).

[B19] Joyner M. J. Coyle E. F. (2008). Endurance exercise performance: the physiology of champions. J. Physiol. 586, 35–44. 10.1113/jphysiol.2007.143834 17901124 PMC2375555

[B20] Malm C. B. Khoo N. S. Granlund I. Lindstedt E. Hult A. (2016). Autologous doping with cryopreserved red blood cells - Effects on physical performance and detection by multivariate statistics. PLoS ONE. 11:e0156157. 10.1371/journal.pone.0156157 27284981 PMC4902314

[B21] Manchado C. Tortosa-Martinez J. Vila H. Ferragut C. Platen P. (2013). Performance factors in women's team handball: physical and physiological aspects–a review. J. Strength Cond. Res. 27, 1708–1719. 10.1519/JSC.0b013e3182891535 23439330

[B22] Marchand A. Buisson C. Martin L. Martin J. A. Molina A. Ressiot D. (2017). Report on an anti-doping operation in Guadeloupe: high number of positive cases and inferences about doping habits. Drug Test. Anal. 9, 1753–1761. 10.1002/dta.2185 28296276

[B23] McArdle W. D. Katch F. I. Katch V. L. (2019). Energy for Physical Activity Exercise Physiology: Energy, Nutrition, and Human Performance. 7th Edn. Philadelphia, PA: Lippincott Williams and Wilkins (2010). p. 106–247.

[B24] Morkeberg J. Saltin B. Belhage B. Damsgaard R. (2009). Blood profiles in elite cross-country skiers: a 6-year follow-up. Scand. J. Med. Sci. Sports. 19, 198–205. 10.1111/j.1600-0838.2008.00770.x 18282224

[B25] Mullen J. Borjesson A. Hopcraft O. Schulze J. J. Ericsson M. Rane A.. (2018). Sensitivity of doping biomarkers after administration of a single dose testosterone gel. Drug Test. Anal. 10, 839–848. 10.1002/dta.2341 29150907

[B26] Nemoto I. Iwaoka K. Funato K. Yoshioka N. Miyashita M. (1988). Aerobic threshold, anaerobic threshold, and maximal oxygen uptake of Japanese speed-skaters. Int. J. Sports Med. 9, 433–437. 10.1055/s-2007-1025046 3253234

[B27] Pound R. W.. (2015). Independent Commission Report #1.

[B28] Pyne D. B. Sharp R. L. (2014). Physical and energy requirements of competitive swimming events. Int. J. Sport Nutr. Exerc. Metab. 24, 351–359. 10.1123/ijsnem.2014-0047 25029351

[B29] Robinson N. Sottas P. E. Schumacher Y. O. (2017). The athlete biological passport: how to personalize anti-doping testing across an athlete's career? Med. Sport Sci. 62, 107–118. 10.1159/000460722 28578329

[B30] Rubana I. E. Aulik I. V. (1989). Buffer function of hemoglobin. Sports Med Train. Rehabil. 1, 125–126. 10.1080/15438628909511862

[B31] Sgro P. Sansone M. Sansone A. Romanelli F. Di Luigi L. (2018). Effects of erythropoietin abuse on exercise performance. Phys. Sportsmed. 46, 105–115. 10.1080/00913847.2018.1402663 29113535

[B32] Slimani M. Znazen H. Miarka B. Bragazzi N. L. (2019). Maximum oxygen uptake of male soccer players according to their competitive level, playing position and age group: implication from a network meta-analysis. J. Hum. Kinet. 66, 233–245. 10.2478/hukin-2018-0060 30988857 PMC6458571

[B33] Solheim S. A. Bejder J. Breenfeldt Andersen A. Morkeberg J. Nordsborg N. B. (2019). Autologous blood transfusion enhances exercise performance-strength of the evidence and physiological mechanisms. Sports Med. Open 5:30. 10.1186/s40798-019-0204-1 31286284 PMC6614299

[B34] Solheim S. A. Morkeberg J. Dehnes Y. Hullstein I. Juul A. Upners E. N.. (2020). Changes in blood parameters after intramuscular testosterone ester injections - Implications for anti-doping. Drug Test. Anal. 12, 1019–1030. 10.1002/dta.2803 32307878

[B35] Sottas P. E. Robinson N. Fischetto G. Dolle G. Alonso J. M. Saugy M. (2011b). Prevalence of blood doping in samples collected from elite track and field athletes. Clin. Chem. 57, 762–769. 10.1373/clinchem.2010.156067 21427381

[B36] Sottas P. E. Robinson N. Rabin O. Saugy M. (2011a). The athlete biological passport. Clin. Chem. 57, 969–976. 10.1373/clinchem.2011.162271 21596947

[B37] Sottas P. E. Robinson N. Saugy M. (2010). The athlete's biological passport and indirect markers of blood doping. Handb. Exp. Pharmacol. 195, 305–326. 10.1007/978-3-540-79088-4_14 20020371

[B38] Stray-Gundersen J. Videman T. Penttila I. Lereim I. (2003). Abnormal hematologic profiles in elite cross-country skiers: blood doping or? Clin. J. Sport Med. 13, 132–137. 10.1097/00042752-200305000-00002 12792206

[B39] Tesch P. A.. (1995). Aspects on muscle properties and use in competitive Alpine skiing. Med. Sci. Sports Exerc. 27, 310–314. 10.1249/00005768-199503000-00004 7752855

[B40] van der Zwaard S. Brocherie F. Jaspers R. T. (2021). Under the hood: skeletal muscle determinants of endurance performance. Front. Sports Act Living 3:719434. 10.3389/fspor.2021.719434 34423293 PMC8371266

[B41] WADA (2014). World Anti-Doping Code 2021. Montreal, QC: World Anti-Doping Agency (2021). p. 184.

[B42] WADA (2020). Technical Document for Sport Specific Analysis. World Anti-Doping Agency (2020). p. 25.

[B43] WADA (2021a). Guidelines for Implementing an Effective Testing Program. World Anti-Doping Agency (2021). p. 84.

[B44] WADA (2021b). International Standard for Testing, and Investigations World Anti-Doping Agency p. 83.

[B45] WADA (2022). Testing Figures World Anti-Doping Agency. Available online at: https://www.wada-ama.org/en/resources/anti-doping-stats/anti-doping-testing-figures-report (accessed May 27, 2022).

[B46] Zorzoli M. (eds). (2012). Blood Monitoring in Anti-Doping Setting. In: *Recent Advances In Doping Analysis Sport und Buch Strauß* (2005). Köln.

[B47] Zorzoli M. Pipe A. Garnier P. Y. Vouillamoz M. Dvorak J. (2014). Practical experience with the implementation of an athlete's biological profile in athletics, cycling, football and swimming. Br. J. Sports Med. 48, 862–866. 10.1136/bjsports-2014-093567 24648438

[B48] Zorzoli M. Rossi F. (2010). Implementation of the biological passport: the experience of the International Cycling Union. Drug Test. Anal. 2, 542–547. 10.1002/dta.173 21204287

[B49] Zorzoli M. Rossi F. (2012). Case studies on ESA-doping as revealed by the biological passport. Drug Test. Anal. 4, 854–858. 10.1002/dta.1340 22514122

